# Design and Development of Learning Management System Huemul for Teaching Fast Healthcare Interoperability Resource: Algorithm Development and Validation Study

**DOI:** 10.2196/45413

**Published:** 2024-01-29

**Authors:** Sergio Guinez-Molinos, Sonia Espinoza, Jose Andrade, Alejandro Medina

**Affiliations:** 1 School of Medicine Universidad de Talca Talca Chile; 2 Interoperability Area National Center for Health Information System Santiago Chile

**Keywords:** interoperability, health information system, Health Level Seven International, HL7, Fast Healthcare Interoperability Resource, FHIR, certification, training, interoperable, e-learning, application programming interface, API

## Abstract

**Background:**

Interoperability between health information systems is a fundamental requirement to guarantee the continuity of health care for the population. The Fast Healthcare Interoperability Resource (FHIR) is the standard that enables the design and development of interoperable systems with broad adoption worldwide. However, FHIR training curriculums need an easily administered web-based self-learning platform with modules to create scenarios and questions that the learner answers. This paper proposes a system for teaching FHIR that automatically evaluates the answers, providing the learner with continuous feedback and progress.

**Objective:**

We are designing and developing a learning management system for creating, applying, deploying, and automatically assessing FHIR web-based courses.

**Methods:**

The system requirements for teaching FHIR were collected through interviews with experts involved in academic and professional FHIR activities (universities and health institutions). The interviews were semistructured, recording and documenting each meeting. In addition, we used an ad hoc instrument to register and analyze all the needs to elicit the requirements. Finally, the information obtained was triangulated with the available evidence. This analysis was carried out with Atlas-ti software. For design purposes, the requirements were divided into functional and nonfunctional. The functional requirements were (1) a test and question manager, (2) an application programming interface (API) to orchestrate components, (3) a test evaluator that automatically evaluates the responses, and (4) a client application for students. Security and usability are essential nonfunctional requirements to design functional and secure interfaces. The software development methodology was based on the traditional spiral model. The end users of the proposed system are (1) the system administrator for all technical aspects of the server, (2) the teacher designing the courses, and (3) the students interested in learning FHIR.

**Results:**

The main result described in this work is Huemul, a learning management system for training on FHIR, which includes the following components: (1) Huemul Admin: a web application to create users, tests, and questions and define scores; (2) Huemul API: module for communication between different software components (FHIR server, client, and engine); (3) Huemul Engine: component for answers evaluation to identify differences and validate the content; and (4) Huemul Client: the web application for users to show the test and questions. Huemul was successfully implemented with 416 students associated with the 10 active courses on the platform. In addition, the teachers have created 60 tests and 695 questions. Overall, the 416 students who completed their courses rated Huemul highly.

**Conclusions:**

Huemul is the first platform that allows the creation of courses, tests, and questions that enable the automatic evaluation and feedback of FHIR operations. Huemul has been implemented in multiple FHIR teaching scenarios for health care professionals. Professionals trained on FHIR with Huemul are leading successful national and international initiatives.

## Introduction

A critical requirement for universal access to health is to have interconnected and interoperable health systems that guarantee effective and efficient access to quality data, strategic information, and tools for decision-making and people’s well-being [1]. One of the most relevant areas in medical informatics is the interoperability between health information systems. The interoperability eliminates duplication and errors in health data. For this reason, health informatics professionals must be educated about the benefits of interoperable systems. Therefore, strategic education on eHealth and interoperability standards is needed to enable health care professionals to make informed decisions [2].

The Fast Healthcare Interoperability Resource (FHIR) is an interoperability standard used in health information technology, introduced in 2011 by the Standard Developing Organization Health Level Seven International (HL7) [3]. FHIR is based on previous HL7 standards (HL7 versions 2 and 3 and Clinical Document Architecture) and combines their advantages with established modern web technologies such as a Representational State Transfer (REST) architecture [4], application programming interface (API), XML, JSON formats, and authorization tools (Open Authorization). The main idea behind FHIR was to build a set of resources and develop http-based REST APIs to access and use these resources. FHIR uses components called resources to access and perform operations on patient health data at the granular level [5,6].

The adoption of FHIR in health information systems by developers and companies has grown in recent years with multiple applications in various fields [5,7-9]. Thus, FHIR is positioned as an interoperability standard that is easy to understand by nontechnology professionals, with fast learning curves that minimize the development time of applications and new tools. In addition, its technological core is aligned with the latest architectures and web standards that allow the development of open APIs, which facilitates interoperability between systems [10].

Teaching and learning interoperability standards, particularly FHIR, within digital health education programs have been oriented more toward delivering content, presentations, and audiovisual material, considering the solution of practical problems separately [2]. Continuously emerging new technologies (synchronous and asynchronous) promise new and improved experiences for individual users but often bring new challenges [11].

The existing learning management systems (LMSs) are oriented to support cross-cutting activities (forums, chat, and content uploading) with content delivery (videos, documents, and links) [12] but not to evaluate REST operations for accessing and using resources. For the use of APIs, some platforms allow interaction with FHIR servers, such as Postman (Postman, Inc) or Insomnia (Kong Inc). However, they cannot create content, manage questions, automatically evaluate the response, or provide feedback but only act as an interface between the user and the FHIR server.

The configuration currently used to teach FHIR is to publish the contents in an LMS or website and, for practice, use tools such as Postman [13,14] without the possibility of having automatic feedback and correction of the activities. The results of the practical exercises must be uploaded as a document to the LMS, with written create, read, update, and delete (CRUD) operations and server response in plain text. The teacher must review them, which makes it challenging to implement workshops with many questions for large groups of students. Other websites offer the opportunity to learn FHIR with guides and theoretical content, such as Simplifier (Firely Corporation). It should be noted that Simplifier is a platform for building FHIR implementation guides. It does not claim to be an LMS or to manage courses.

There is currently no LMS for training on FHIR that allows problem-oriented assessment and practice of web-based CRUD operations. Practice is essential to learn FHIR; therefore, a problem-oriented platform is necessary, allowing the creation and administration of practical courses (where a problem is presented) with different levels of complexity and for multiple professionals (clinicians, engineers, and technicians). In addition, each course should be associated with a set of exercises, which the students must answer with CRUD operations (eg, create a patient with the data given in the description or modify the patient information with the new phone number provided). The platform should automatically evaluate these answers, and feedback should be provided to guide each question’s achievement (or nonachievement). This would help generate an extensive repository of massive web-based training programs focused on specific problems, where students must practice as requested. The lack of such platforms has motivated the interoperability team of the National Center for Health Information System (CENS) [15] to design a tool capable of automatically teaching and evaluating FHIR.

In this sense, our goal was to develop an API that allows us to integrate and communicate a set of loosely coupled modules that enable teachers to manage FHIR training programs, designing courses, questions, and scenarios. In addition, learners can interact through a web client for self-learning sessions, where the API, in conjunction with an assessment engine, provides feedback for each attempt the learner makes. This undoubtedly streamlines the self-learning process and automates the correction of hundreds of CRUD operations and the submission of learner responses within a context that the platform delivers.

The design and development of a platform called Huemul support the creation of courses associated with multiple questions (which expect a CRUD operation as an answer), automate the evaluation of the responses, and provide automatic feedback to the students in each exercise. We have also created an administrator that allows us to create and manage courses, questions, and users.

## Methods

### Study Design

The e-learning system requirements for teaching FHIR were collected through interviews with experts involved in academic and professional activities (universities and health institutions). The interviews were semistructured, recording and documenting each meeting. In addition, we used an ad hoc instrument to register and analyze all the needs to elicit the requirements.

The CENS academic committee, formed by 5 senior biomedical informatics researchers (3 engineers: 2 biomedical and 1 informatics and 2 medical doctors), was the initial core of experts consulted. In another focus group, engineers from the interoperability area of CENS, experts in FHIR, were consulted. They presented their requirements and needs to automate both the deployment and evaluation of the different interoperability challenges organized by CENS, where the need to register, quantify, and evaluate the hundreds of requests sent by the participants to the server was a problem when assessing their tests. These interoperability events were part of Chile’s CENS human capital training program.

Both academics and CENS engineers were interviewed with the following questions: Do you think a platform for teaching HL7 FHIR is necessary? What functions should it have? What non-functional requirements do you think are essential for the platform? For more details, see Multimedia Appendix 1.

Finally, the students (engineers from health institutions) were consulted on the platform’s functionality, modules, and usability in the first application of the pilot. A small instrument with 5 questions on a Likert scale (scale of 1-5) was applied to assess the application and the proposed modules, considering the user interface, quality of feedback, response times, quality of the content, and the response console. In addition, 2 open-ended questions were asked about the advantages and disadvantages of the platform.

The focus group sessions were transcribed, the topics of interest were categorized (user profile, usability, perceptions of use, and design), the patterns present were identified and interpreted, and the information obtained was triangulated with the available evidence. This analysis was carried out with Atlas-ti software (Scientific Software Development GmbH). With this information, the final prototype and the website for its deployment were designed.

End users are classified according to the following profiles: (1) system administrator in charge of the deployment and administration of the modules, client, and all technical aspects of the server; (2) professor who designs the course and describes the clinical context and associated questions; and (3) students in charge of accessing the client to answer questions about the course they are enrolled in.

### Requirements

The system design requirements were divided into functional and nonfunctional (Textbox 1). The system development aimed to support the functional requirements to run e-learning sessions for FHIR courses. Regarding the nonfunctional requirements, security and usability are essential to design functional and secure interfaces by considering technological aspects, learner interactions, and instructional design [16,17] (Table 1). For more details, see Multimedia Appendix 1.

**Functional requirements to design the system for teaching FHIR (Fast Healthcare Interoperability Resource).**

**1. Test and question manager:**

- Users’ management
- FHIR create, read, update, and delete (CRUD)-oriented test management
- FHIR CRUD operations
- CRUD courses
- Create and manage a database with questions, tests, and courses

For an FHIR test (where the context and the problem are explained), examples of questions could be:

- Create the patient with the information given in the description
- Create a medical encounter
- Modify the phone number and address of the doctor
- Delete the patient

**2. Application Programming Interface (API) for orchestrating components:**

- Users’ authentication management
- Call up tests and questions
- Validate user answers
- Save user answers
- Execute FHIR CRUD operation on the server

**3. Test evaluator:**

- Evaluate answers
- Compare questions and answers
- Build resources with the HAPI FHIR library
- Validate resources with standard

The expected answer should be a CRUD operation for a FHIR test (where the context and the problem are explained). For example, for the creation of a patient, the student must complete the following:

- Method for creating a FHIR resource (post)
- [FHIR Endpoint]/patient (URL server and resource name)
- Patient data (JSON format; patient information)

**4. Client application:**

- Create responsive front end
- Communicate using the Huemul API
- Decoupled other components

**Table 1:** Tools, libraries, and relation with each software component.

Development area and tools or libraries				Related component							
				Engine	Admin		API^a^		Client		FHIR^b^ server
**Environment**											
	NetBeans			✓	✓		✓		✓		✓
	IntelliJ CE			✓	✓		✓		✓		✓
**Backend**											
	**Python**										
		Python 3.6			✓	✓					
		Celery			✓						
		Django 3.1			✓	✓					
		Django DRF 3.1				✓					
	**Java**										
		OpenJDK 11	✓							✓	
		Apache Maven	✓							✓	
		Apache Tomcat 9	✓							✓	
		HapiFhir 5.3	✓							✓	
**Front end**											
	Bootstrap 4.3								✓		
	jQuery 3.1.1								✓		
**Deployment**											
	Docker										
	Docker Compose										
**Database**											
	MySQL 5.7			✓	✓		✓		✓		✓

^a^API: application programming interface.

^b^FHIR: Fast Healthcare Interoperability Resource.

### Software Development Methodology

The development methodology was based on the traditional spiral model. The spiral development model starts with a small set of requirements and goes through each development iteration for that set of requirements. Then, the development team adds functionality for the additional requirement in ever-increasing spirals until the application is ready for the production phase [18].

Each iteration has objectives related to the evolution of the components to be developed:

1. Modeling and management: in the first iteration, a functional database model was generated with the objective that it can support the definition of models related to tests, users, questions, and courses and the creation of FHIR learning tests. In addition, an administration application (Huemul Admin) was created to maintain the generated models. Once the model was built, a REST API (Huemul API) was developed to consult the information.
2. Improvements to the data model and API: in the second iteration, improvements to the model were included with the analysis of the previous iterations, authentication and security features of the REST API, and the creation of a web client (Huemul Client) for the consumption and interaction of the REST API.
3. Response processing and evaluation: in the third iteration, models for response processing are included, an interface for sending responses to the web client is added, and an engine (Huemul Engine) for response evaluation is created. The administrator creates a test planning mechanism, setting start and end times.
4. Functional improvements and feedback: in the fourth iteration, modifications are introduced in the processing of answers, feedback in case of incorrect answers, and the enabling of a natural resource query interface.

Each developed component has a set of tools described in Table 1, the languages used are Python (Python Software Foundation) and Java (Oracle Corporation) in the backend, and all interaction between components involves using a REST API. In addition, the front end group has some traditional libraries for client development, as it uses another API to consume resources independently and does not restrict alternative clients.

Three full-time computer engineers and the leader of the CENS interoperability area worked on the platform to create the software. It took 6 months to develop the prototype and 1 month to make modifications during the pilot implementation.

#### Ethical Considerations

It should be noted that this research complied with ethical standards in accordance with the Declaration of Helsinki (updated in 2013).

## Results

### Overview

Huemul has 4 components that were designed and named considering the functional and nonfunctional requirements. Therefore, the following modules are necessary to develop a scalable and robust system:

1. Huemul Admin: web application to create users, tests, questions, and scores.
2. Huemul API: communication between different components of Huemul (FHIR server, client, and engine).
3. Huemul Engine: answers evaluation to identify differences and validate responses.
4. Huemul Client: web application for users to show the test and questions.

**Figure 1:**
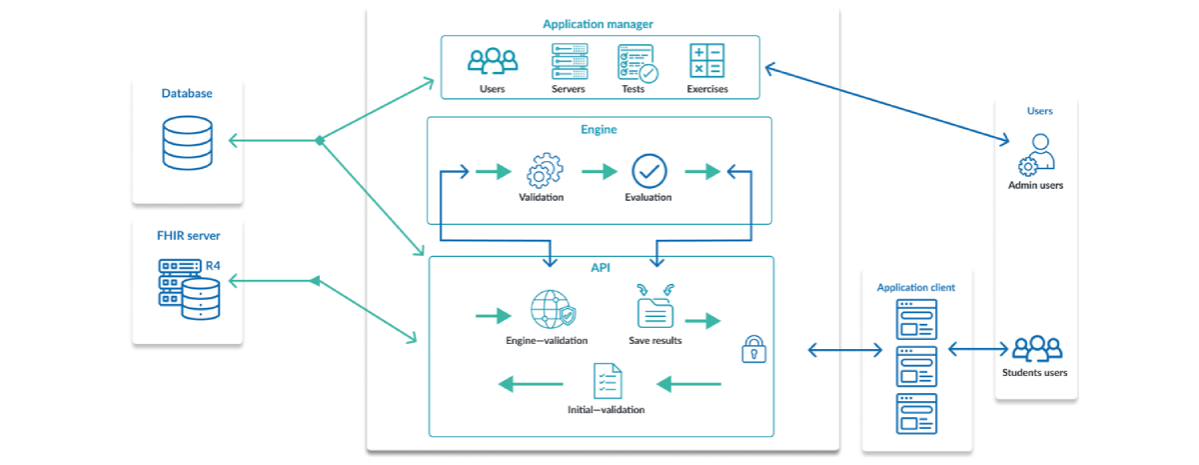
The system architecture of Huemul with the components and their relations. API: application programming interface; FHIR: Fast Healthcare Interoperability Resource.

The architecture of the developed system allows for the separation into different layers. For example, the software was built under the Model-View-Controller architecture [19] to separate the views from the data model and the business logic (Figure 1). Furthermore, since usability is one of the most important nonfunctional requirements, views use web technologies, such as HTML5, JavaScript, and CSS3, to ensure access to different web browsers.

The front end can display the courses created and managed by the administration component, where the users can answer each question. In the business-oriented layer, Huemul API interconnects with the validation engine and communicates the user’s answers to this engine, which oversees validating and reviewing their structure and content. The API is Huemul’s communication core. Once a user’s response has been validated, it connects the operation with the backend (HAPI FHIR server) and communicates the result to the client.

### Huemul Admin

**Figure 2:**
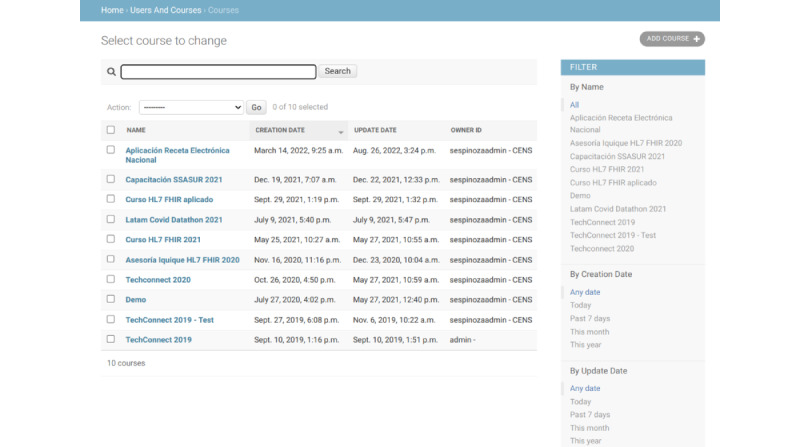
Huemul Admin component with active FHIR courses in the platform. CENS: National Center for Health Information System; FHIR: Fast Healthcare Interoperability Resource; HL7: Health Level Seven International.

The admin component was developed as a web application to create users, tests, and questions with associated test scores. This component is decoupled from the overall system architecture, providing independence and modularity. Figure 2 shows a set of screenshots with the main functionalities of the Huemul Admin component. It shows the questions created, associated FHIR servers, tests, users, and courses. Each mentioned element can be modified and associated with generating modular courses that are easy to administer.

It is essential when creating a course to situate the clinical scenario within a context (outpatient, emergency, inpatient, and home). This will help health professionals, who are learning about interoperability, to better design the necessary resources, and CRUD operations required to solve the problems presented.

### Huemul API

**Figure 3:**
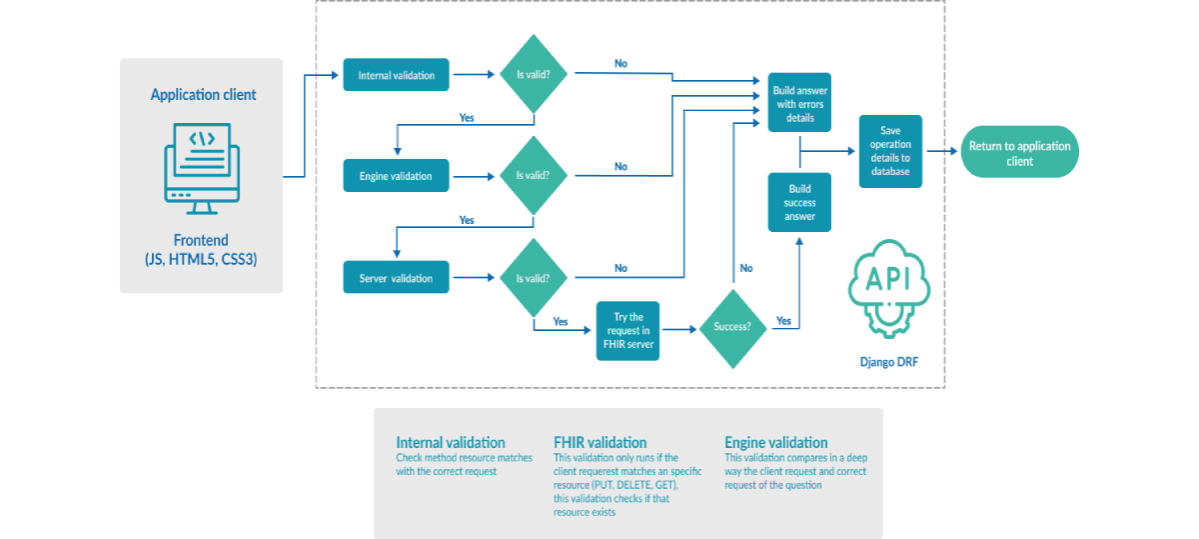
Huemul API component that communicates with all the components of the system. API: application programming interface; FHIR: Fast Healthcare Interoperability Resource.

The core of the communication is Huemul API. This API communicates the different components of Huemul (FHIR server, client, and evaluation engine), orchestrating the whole system. An essential task of the API is communicating between the client and the evaluation engine. The test evaluation process begins when the learner sends an answer through the Huemul client application until the response is received. Specifically, the steps are as follows (Figure 3):

1. Send a request from the client: the student sends the response through the client application.
2. Internal validation: the API performs basic validations of the request sent from the client. It validates the server URL, the headers, and the body of the JSON content.
3. Engine validation: performs a full validation by comparing the answer sent by the student with the expected answer configured when creating the question.
4. Evaluation response: once all the validations have been carried out, the result is delivered, either a successful or unsuccessful comparison.
5. FHIR request: once the expected response has been validated against the one sent, if the evaluation in the engine was successful, the student’s response is sent to the corresponding FHIR server to be saved.
6. FHIR response: the FHIR server receives the request, processes it, and assigns a destination variable to the resource to identify the student who sends the response and responds to the API.
7. Build success answer: if the response from the FHIR server is successful, the API constructs the response with the summary of the validation process, evaluation, and result from the FHIR server, which will be sent to the client application.
8. Response: the API sends the answer to the client application so that the result of its submission is displayed on the screen to the learner.

### Huemul Engine

**Figure 4:**
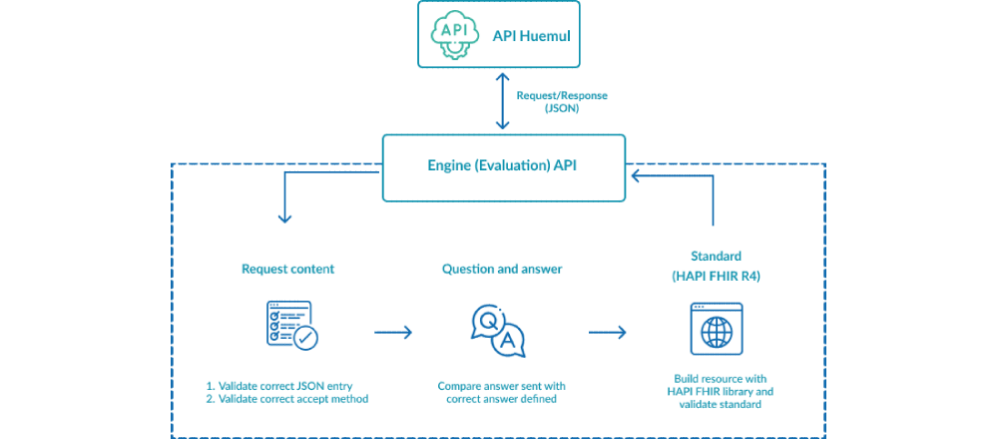
Huemul Engine component that validates, evaluates, and builds the response. API: application programming interface; FHIR: Fast Healthcare Interoperability Resource.

This component has the function of response evaluation, for which it evaluates 2 responses, the expected response and the user’s response. The processing comprises 3 subprocesses to finally have an evaluation result that allows us to assess if the answer is correct or to assess the percentage of completeness (Figure 4).

A FHIR request, by definition, contains the following elements to be assessed:

- Base URL of the FHIR server.
- Path of the resource or query to be made to the server.
- The header of the requested content is JSON or XML.
- The body of the resource is JSON or XML format if, in case, REST methods require a body; otherwise, the body will not have information for the request.

The methods accepted to create a question are POST, PUT, GET, and DELETE.

### Huemul Client

**Figure 5:**
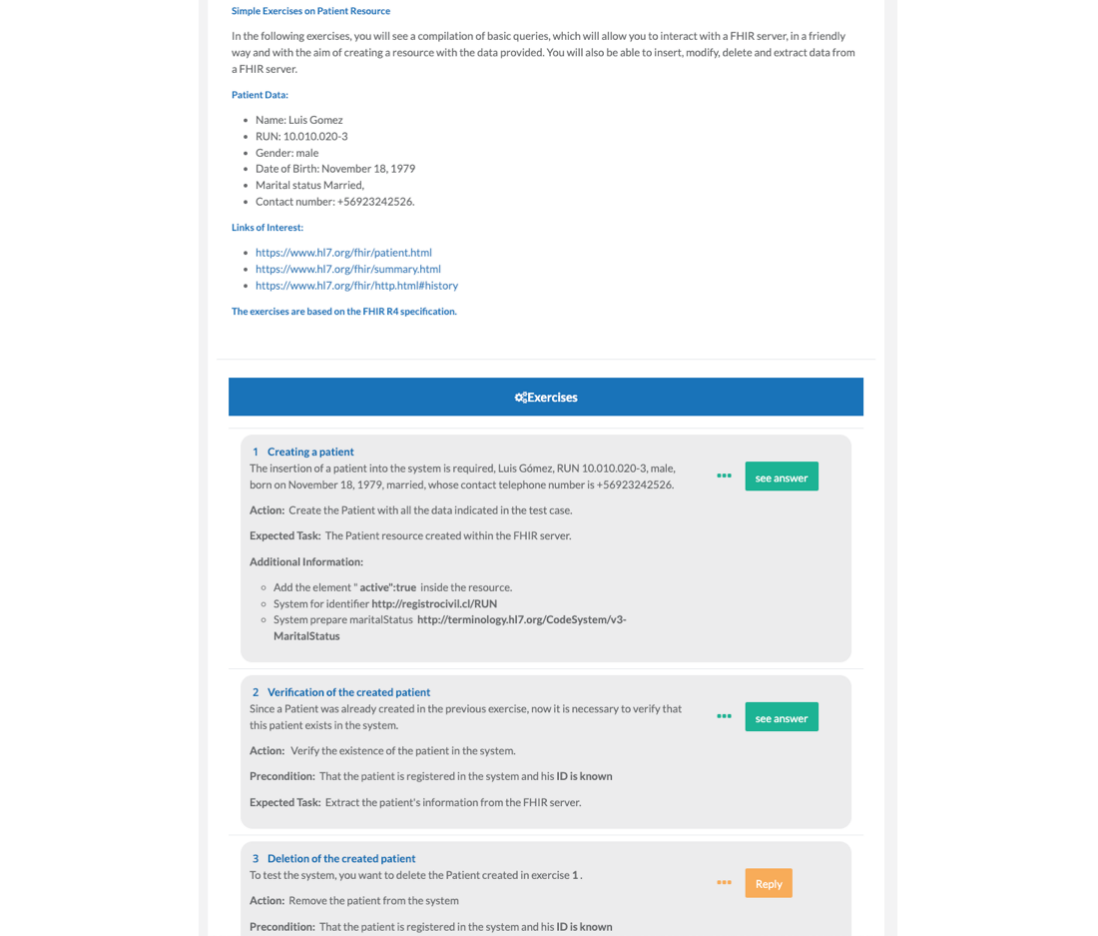
Huemul Client with a test consisting of an explanation of the scenario and associated questions. FHIR: Fast Healthcare Interoperability Resource.

Huemul provides a web client for users, allowing them to display the test and the questions, and is the interface with the platform. For example, on the screen for sending the answer, the question statement and essential information for answering (action, precondition, expected task, etc) are presented; there is also a button to visualize the description of the scenario, and below in notifications, the platform gives feedback to the user to improve and correct the answers (Figure 5). For more details, see Multimedia Appendix 2.

When the user enters a course, the client presents the complete scenario, including information relevant to the test. Below is a list of the exercises to be answered; each activity has an associated answer button with different colors.

- Orange button: exercise active but still needs to be answered.
- Green button: exercise with the correct answer.
- Red button: exercise with the wrong answer.

### Initial Evaluation of Huemul Use

In early 2020, we conducted a pilot project in which we invited 20 health care professionals from different national institutions (10 systems development, 3 physicians, 4 computer scientists, and 3 nurses). They were students in a pilot course that presented a clinical situation and had to answer the questions through CRUD operations with HL7 FHIR. Once the course was completed, we gave them 5 questions. The questions had 5 scores according to the Likert scale for quality: 1=very poor, 2=poor, 3=fair, 4=good, and 5=excellent.

Each question focused on evaluating aspects related to the following five dimensions:

1. End-user interface: the platform is accessible and attractive for students.
2. Quality of response: feedback provided by the platform was helpful.
3. Response times: platform response times are adequate.
4. Quality of content: course description and questions are adequate.
5. Response console: response console is intuitive and easy to use.

In addition, we incorporated 2 open-ended questions that inquired about the advantages and disadvantages of the platform. The most rates of the dimensions scored on average above 4 (response times=4.9, quality of content=5, and response console=4.6). The only dimensions that did not cut above 4 on average were end-user interface and quality of feedback, with averages of 3.4 and 3.0, respectively.

This was consistent with the qualitative analysis of the open-ended questions, where students rated the content, questions, response times, and the working console positively. In general, they expressed the platform’s usefulness for self-study of FHIR. However, the usability was criticized concerning the navigation between the questions and the test, the font and size of the text, and the lack of information to support formatting.

Currently, Huemul has the following usage statistics:

- Users: 416 students with one or more courses in the platform.
- Courses: 10 courses.
- Tests: 60 tests.
- Questions: 695 questions (431 used and 264 unused; 572 general questions that can be used by any teacher with a Huemul account and 123 private questions).
- Response rate: 1725 (1666 completed+59 incomplete).

During the last 3 years, including the COVID-19 pandemic, 416 students have answered the same questions to evaluate the platform (with the exact 5 dimensions applied in the 2020 pilot). The evaluation has been good, with slight improvements since the pilot in dimensions 1 and 2. The same open-ended questions were applied in each course. The general comments are good or excellent, with suggestions for improvements, mainly in usability issues. The main criticisms collected in the open questions coincide with the pilot’s answers, making comments for feedback too brief and needing more helpful information to solve the exercise. Another aspect that stands out is usability, color, and font size.

Each comment has helped us to improve, incorporating a graphic designer into the team and improving the navigability of Huemul. In addition, feedback was complemented with templates of the principal associated resources that allow students to learn in a more guided way.

The preliminary impact detected is the increase in interoperability projects associated with FHIR in Chile, where the project leaders are the professionals who participated in the CENS courses with Huemul. In addition, some professionals (clinicians and engineers) were incorporated into the government to work on national strategies linked to FHIR. Other participants were recruited for medical informatics departments in hospitals (both public and private), where they led projects with FHIR.

## Discussion

### Principal Findings

The Huemul FHIR learning platform was designed and developed with loosely coupled components to communicate through a central API orchestrating module communication. This design was fundamental when starting to plan, considering the development of an API rather than a platform. In addition, its decoupling allows the API to interact with different technologies and with other systems and software that students can use while maintaining the independence of the components.

Integrating information dispersed in different systems is a relevant problem in health informatics. Thus, health informatics professionals must strengthen interoperability by learning standards that allow proper use. Currently, the most promising interoperability standard is FHIR. It builds on the concepts of the previous HL7 standards. The main objective of FHIR is to facilitate the implementation of solutions in various contexts: mobile apps, cloud communications, telemedicine, and medical records data sharing, among many others. Therefore, one of its main strengths is its ease of use and better learning curve compared to previous standards; this allows doctors, nurses, and engineers to work together in designing interoperable health care informatics solutions.

To develop competencies in FHIR, Huemul has been fundamental for training professionals in Chile. The CENS [15], with its Health Information Systems (HIS) Reference Competency Model [20], has developed and used it to strengthen and generate competencies in interoperability and standards, especially with HL7 FHIR. The model proposed by CENS brings together consensual knowledge, skills, and attitudes as a reference that guides the training of excellence in biomedical informatics. Moreover, the model drives the design of undergraduate and postgraduate training curricula and establishes common training standards in the country and the region. In addition, it makes it possible to make it evident on what is expected of professionals and technicians in this sector and what is expected of them from the point of view of job opportunities or professional development.

In Chile and Latin America, there is a need for biomedical informatics professionals trained in interoperability and standards for sharing data between HIS [2]. Currently, the demand for professionals with these competencies has increased the digital gap in health and, consequently, has slowed down the changes needed to have a more connected health with robust standards, terminologies, and HIS. Huemul is available for training processes that require new ecosystems and models.

In this context, Huemul is a web application that creates users, tests, and questions to define scores and reviews them automatically in interoperability scenarios with HL7 FHIR. Huemul was the learning platform for Chile’s annual health interoperability meeting in 2020 and 2021 [21]. The interoperability meeting featured 4 sections of HL7 FHIR exercises (patient, diagnostic report, electronic medical prescription, and electronic health record), with 2 levels of complexity: introductory and intermediate. More than 100 participants each year performed hundreds of CRUD operations per exercise, which Huemul reviewed automatically. In addition, Huemul has been the official CENS platform for delivering HL7 FHIR training courses.

As a result, in the last 3 years, more than 400 technicians, engineers, and health professionals interested in learning FHIR from all over the country have been trained so far [20]. Moreover, the CENS academic team generated 10 courses with 60 associated tests. Huemul has made it possible to create a repository with more than 695 questions with different complexity levels. Each applied course has served as feedback, considering that we asked the students about the quality of our platform; considering all the dimensions exposed in the results, the users have a good evaluation of Huemul. We are still working on usability and feedback on the answers; we believe that we must improve and move forward, for example, to mobile devices and expand the content base and application areas.

Most trained professionals are leading interoperability projects with FHIR from the government, universities, and public or private health institutions. CENS continues to support capacity building for both professionals and institutions. In this sense, Huemul is an effective tool to support practical activities, enabling the teaching of FHIR. We expect to continue advancing and complementing Huemul with new functionalities and modules in future work.

### Future Work

Concerning future work, Huemul is currently in the process of redesigning for a 2.0 version that will allow us to incorporate new functionalities:

- Incorporate extensions, profiles, and extended Huemul for more search parameters. This would allow the number of questions, courses, and scenario options to be expanded as well as the complexity of the tests.
- Incorporate multiple choice and true and false questions to prepare for the HL7 FHIR certification examination. Incorporating content questions would give us a robust tool to prepare the CRUD operations in a clinical scenario and the theoretical context that will enable us to schedule examinations and certifications (eg, HL7 FHIR Proficiency examination).
- Create web-based courses with LMSs (for instance, Moodle) and Huemul. Integration with LMS platforms would extend the teaching ecosystem, incorporating content management systems, chat, forums, and all the tools with LMS.
- Incorporate other FHIR servers. Until now, Huemul has been working with HAPI FHIR, which is a complete implementation of the HL7 FHIR standard for health care interoperability in Java [22]. The advantage of having a decoupled system is the ease and modularity of its components. Huemul currently works with HAPI FHIR as a server; however, another server could be incorporated.

Another interesting aspect is evaluating and certifying interoperability levels in health information systems in a natural context [23]. Huemul could extend its applicability to other domains, for example, the assessment of HIS interoperability in hospitals, clinics, and all types of health institutions. Any modifications to its approach would be minimal, as its original 4-component structure would be maintained: Huemul Admin, Huemul API, Huemul Engine, and Huemul Client. The main changes should focus on the client-submitted request evaluation engine, broadening its focus from teaching HL7 FHIR to a more enterprise-based domain.

Considering a detailed systematic evaluation, the platform’s usability is interesting to investigate deeply. Therefore, a study design that allows the application of validated instruments and the collection of information from multiple profiles and professionals is proposed as future work.

### Conclusions

Huemul is the first platform that allows the creation of courses, questions, and scenarios that enable the automatic evaluation and feedback of CRUD operations with HL7 FHIR. Huemul has been implemented and applied in multiple HL7 FHIR teaching scenarios for health care professionals. It has demonstrated its efficiency and effectiveness in courses and massive events, managing hundreds of users and evaluating thousands of answers in these 4 years of application.

Of the 416 students who were trained with Huemul, many are currently leading interoperability projects with HL7 FHIR, both in the government and the private sector, contributing to developing digital health and information systems in Chile.

## Appendix

Multimedia Appendix 1 Huemul functional requirements.

Multimedia Appendix 2 User manual client.

## Abbreviations

API: application programming interface
CENS: National Center for Health Information System
CRUD: create, read, update, and delete
FHIR: Fast Healthcare Interoperability Resource
HIS: Health Information Systems
HL7: Health Level Seven International
LMS: learning management system
REST: Representational State Transfer
