# Supplementary material for: Design and Development of Learning Management System Huemul for Teaching Fast Healthcare Interoperability Resource: Algorithm Development and Validation Study
Source: JMIR Med Educ. 2024 Jan 29;10:e45413. doi: 10.2196/45413 (PMC10862243; doi:10.2196/45413)
Supplement: Multimedia Appendix 1 [file mededu_v10i1e45413_app1.docx]

**Multimedia Appendix 1. Huemul functional requirements.**

**Administrator Component**

| **Modules** | **Functional** |
| --- | --- |
| **1: Answer**  Module that allows you to view the answers and their content grouped by test and/or by user. | - The system must be able to display the answers to the exercises grouped by user, by question and by test. - The system must save all the answers sent from the client, mark the last answer sent and count the answers per exercise. - The system must not allow to modify/delete the answer from the administrator, except for the super administrator user. |
| **2: Server**  Module to manage connections to FHIR servers with which it is possible to interact. | - The system shall allow interaction with FHIR servers in different versions of the HL7 FHIR standard. - The system shall allow the administration of servers (basic connection data) that can be used in testing. |
| **3: Tests**  Module for managing tests, categories, exercises and the application of these tests. | - The system must allow viewing, adding, editing and deleting tests. - The system must allow a test to be reused several times. - The system must allow to view, add and delete exercises within the tests. - The system must allow to define/modify the order of questions and weighting. - The system must allow to view, add, edit and delete categories. |
| **4: Users and Courses**  Module for managing users and participants, courses and the relationship between participants and courses. | - The system must allow to search, list, view, create, edit and delete users, which to be registered must be differentiated by roles and permissions (student, teacher, collaborator, administrator).   The system must allow teacher, collaborator and administrator users to log in and recover their password.   - The system must allow to view, add, edit and delete courses, and block the option to delete users who have not created them or if they contain student answers. - Allow adding, activating and deleting tests from courses. - It must be allowed to add students to a course manually (one by one) and massively from an excel file. |

**API Component**

| **Functional** |
| --- |
| - Must consider the use of token for security. - It must consider requests related to user authentication. - Must support query requests to the FHIR server. - All requests must be made by an authenticated user. - It must allow to obtain a list of the Tests associated to the user. - It must allow to obtain information of a specific Test. - It must allow to obtain a list of the Tests Questions associated to the user. - It must allow to obtain information of a specific question. - It must allow to receive answers to a question of Tests associated to the user. - It must allow to obtain the available servers. - It must allow to obtain the last answer of a question. - It must allow to obtain a summary of all the answers of a Test. - It must allow to obtain the courses associated to the user. - It must allow to obtain information of a specific course. |

**Engine Component**

| **Functional** |
| --- |
| - Validate input structure, that it has JSON format. - Validate JSON structure, that it contains the necessary keys and that the format of each key corresponds to the evaluation. - Compare JSON between the question and the answer. - Allow writing any value as an answer in String variables of a resource, entering "*" as the expected answer. - Validate that the Resource complies with the HL7 FHIR standard. - Calculate the evaluation result of the comparison. - Define error code and write errors as a result of existing. - Build response JSON according to obtained results. |

**App Client Component**

| **Modules** | **Functional** |
| --- | --- |
| **1: Instances**  This screen displays the courses in which the user is associated. | (Post-login screen)   - The user will be able to see the list of all courses (instances) in which he/she is registered in Huemul. - Each of the courses in this screen must have associated its name, description and image corresponding to the information that exists in the administrator and an access button to the list of tests. |
| **2: Test list**  This screen allows you to view the tests associated with the course you have entered. | (Post-course login screen)   - The system must show the list of tests associated to the course. - Each item (course) must have associated name, description, status, closing date and a button to access the details. |
| **3: Test Detail**  This screen displays the details of the test and the questions to answer. | (Test login screen)   - The application must allow the user to see the details of each test (title, scenario and list of exercises). - The list of exercises must have a button associated with the action to be performed. - The button must differentiate the actions that can be performed on the exercise. |
| **4: Question**  Screen that allows you to view the details of the question and the option to answer. | (Question entry screen)   - The application must allow the user to answer each exercise. - The last answer overwrites the previous answer for each exercise. - The application must provide feedback per exercise. |
| **5: General**  Consider some generalities of the application. | - The application must have a screen to perform queries on the server. - The application must have an informative screen of the standard formats that are accepted in the responses. - The application must allow the user to log in, reset password and log out. |
