# Supplementary material for: Design and Development of Learning Management System Huemul for Teaching Fast Healthcare Interoperability Resource: Algorithm Development and Validation Study
Source: JMIR Med Educ. 2024 Jan 29;10:e45413. doi: 10.2196/45413 (PMC10862243; doi:10.2196/45413)

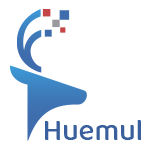


**Multimedia Appendix 2. User manual client.**

**Version 1.0**

**Login**

Log in to <https://app-huemul.cens.cl/login.html> (the web address of the first version of huemul) with the registered credentials.
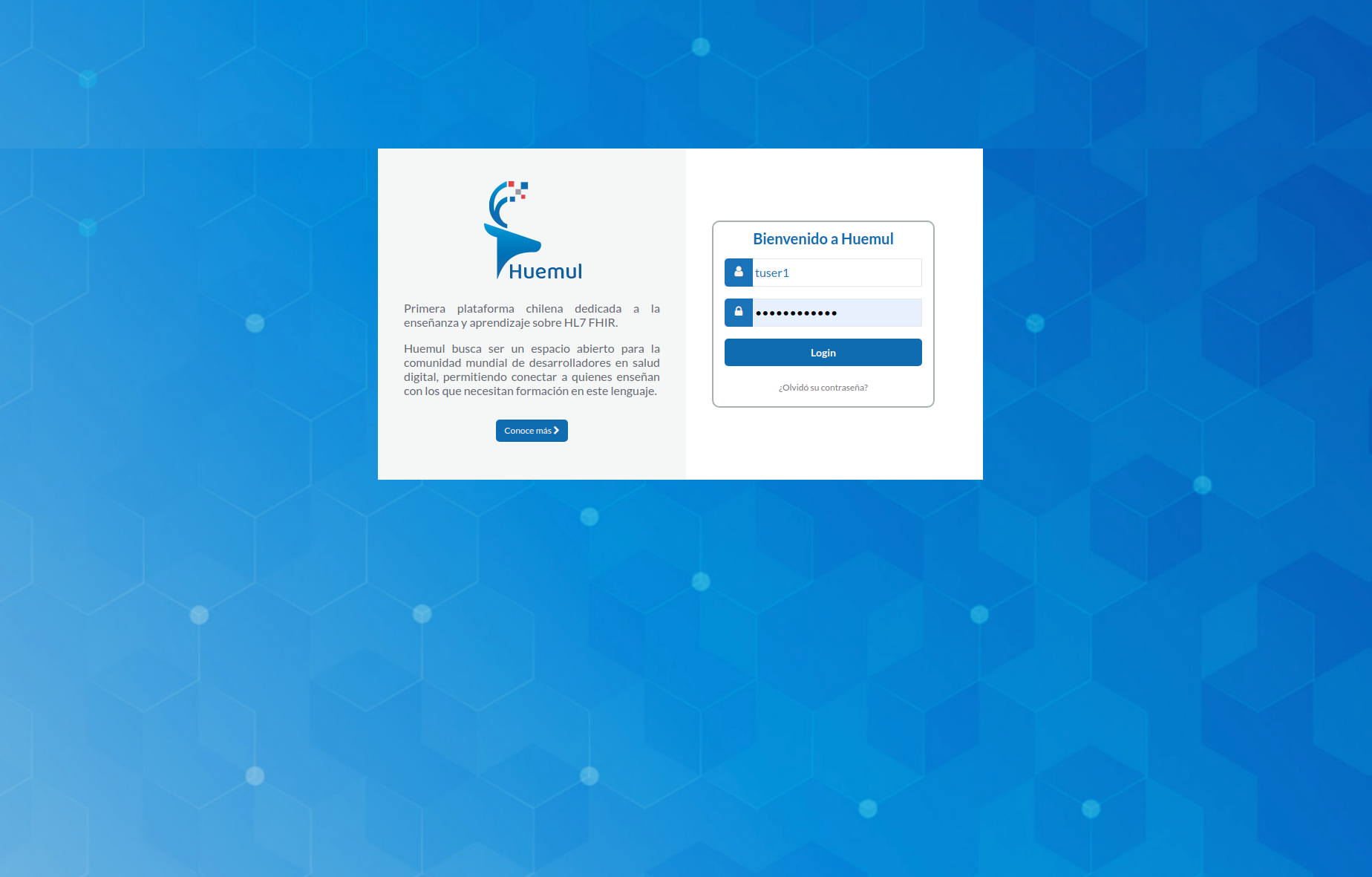


Once logged in, you will see a screen like this.
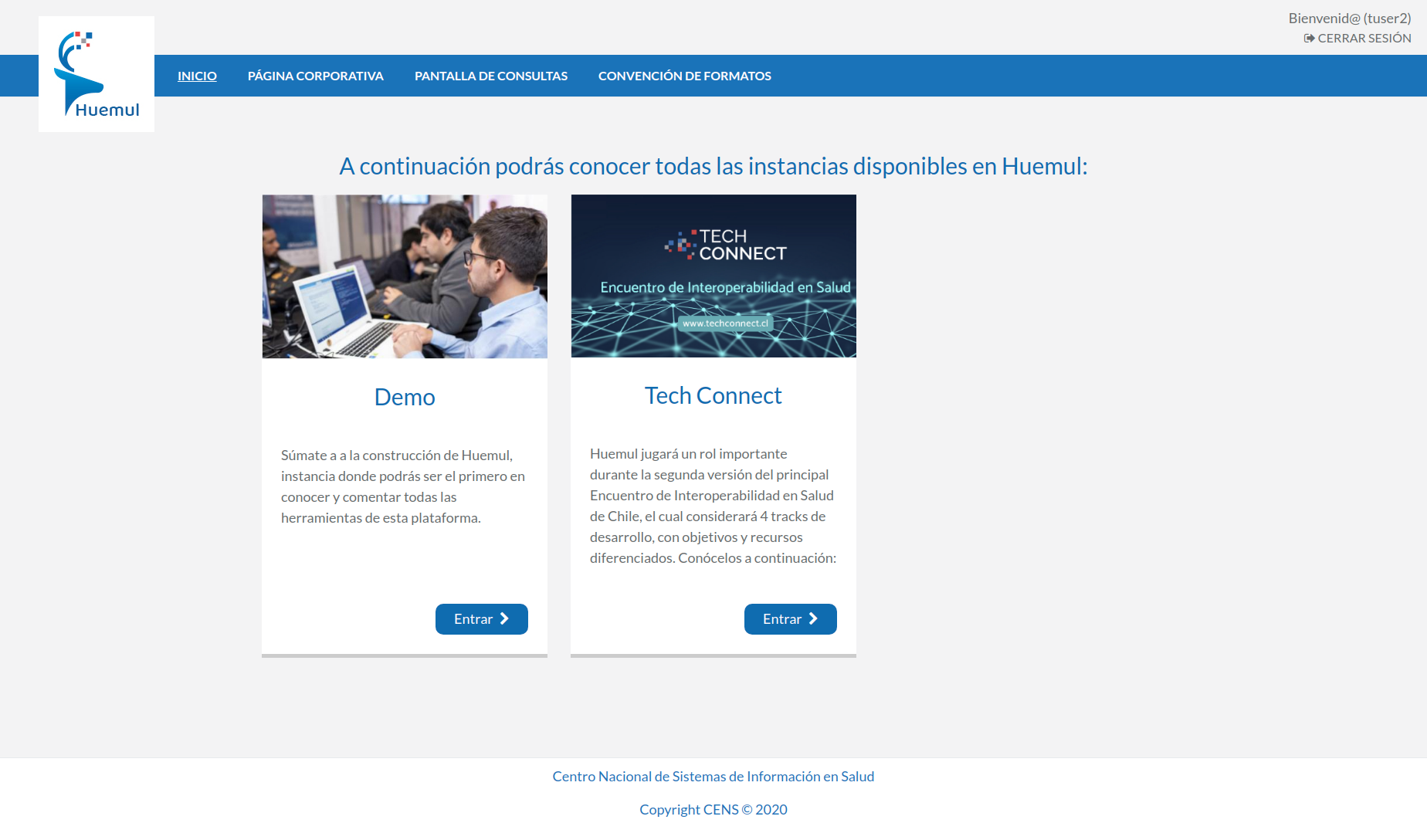


**Log out**

On the top right, you will find the logout button.


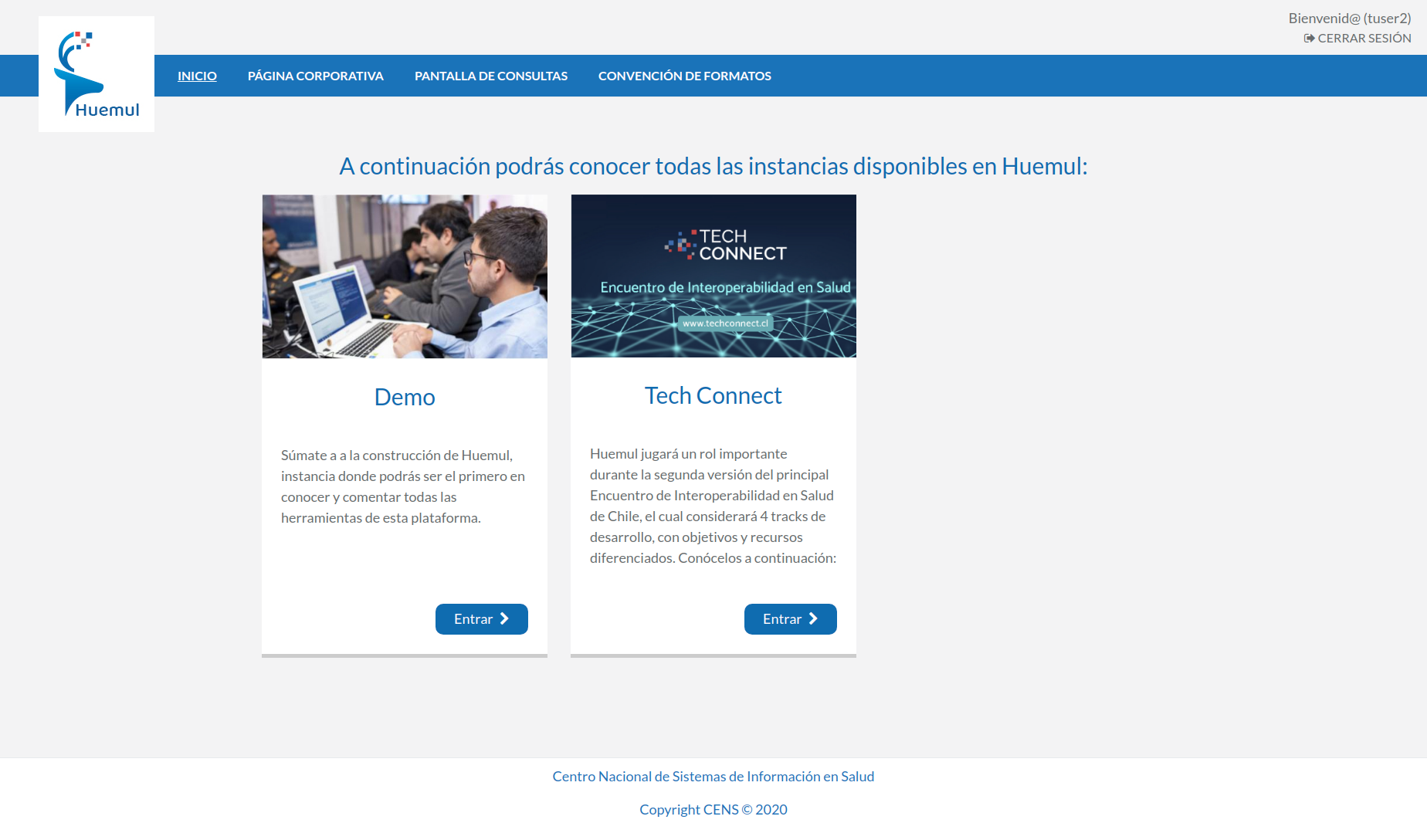


**Top menu**

In the top menu, you will find the following pages:

- Home: Huemul's home page.
- Corporate page: access to the corporate page with Huemul's description.
- Queries screen: It is possible to make queries to the connected FHIR servers on this screen.
- Format convention: access to an external page with the documentation of allowed formats


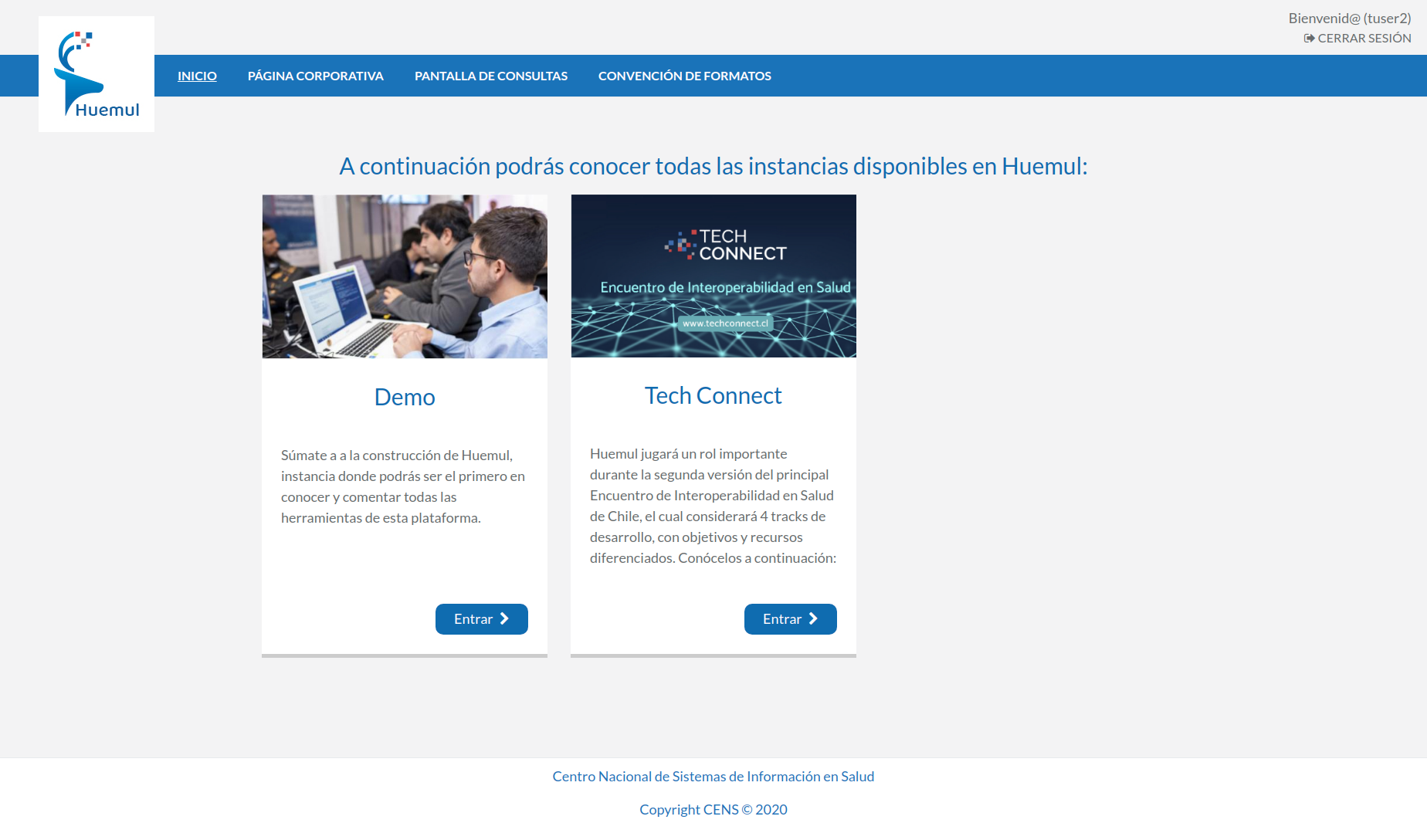


**Home Page**

On the home screen, all Huemul instances are displayed, e.g. demo and tech connect. The user must select the one in which they are registered
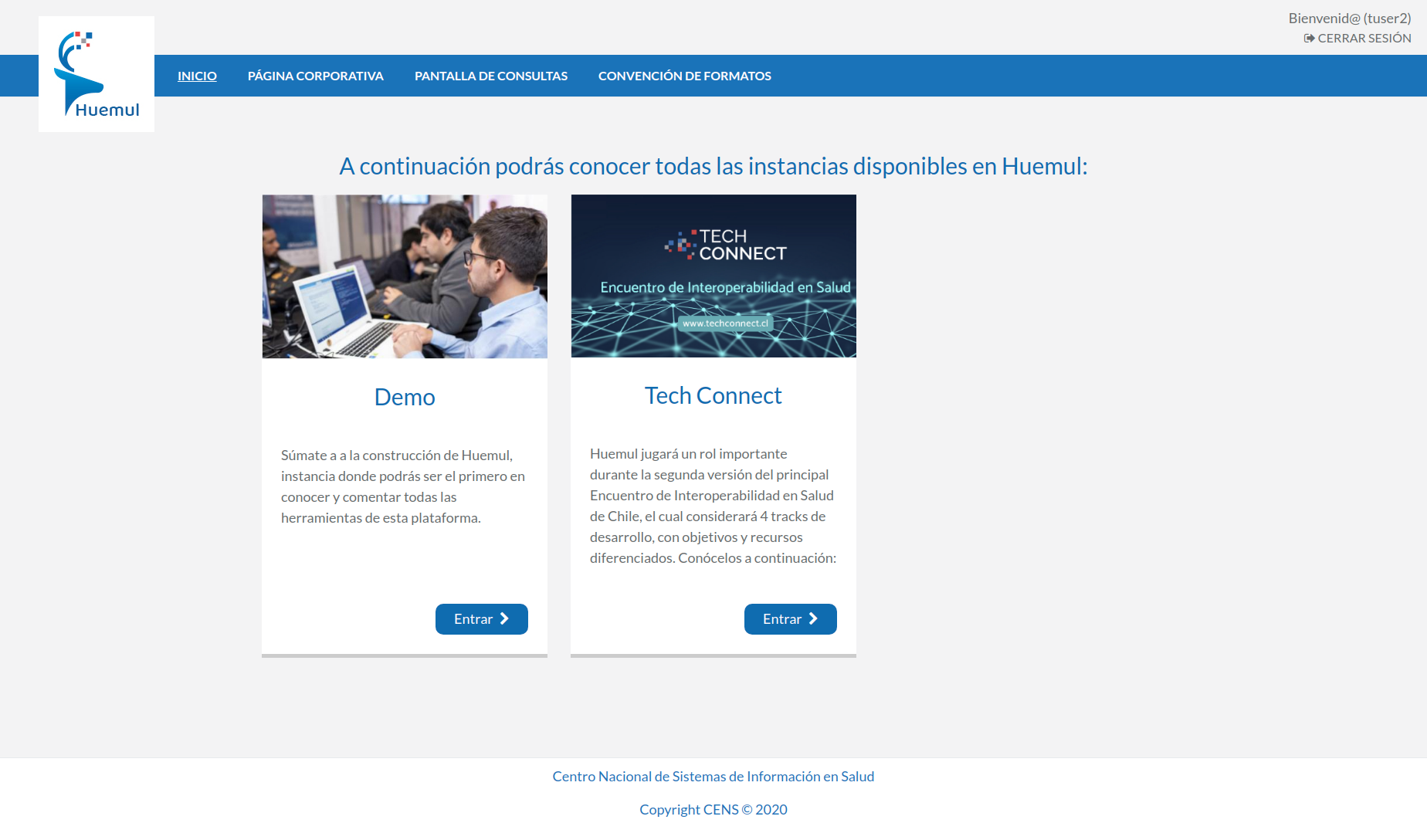
.

**Instances**

- After selecting the instance, the corresponding tests or challenges will be displayed.
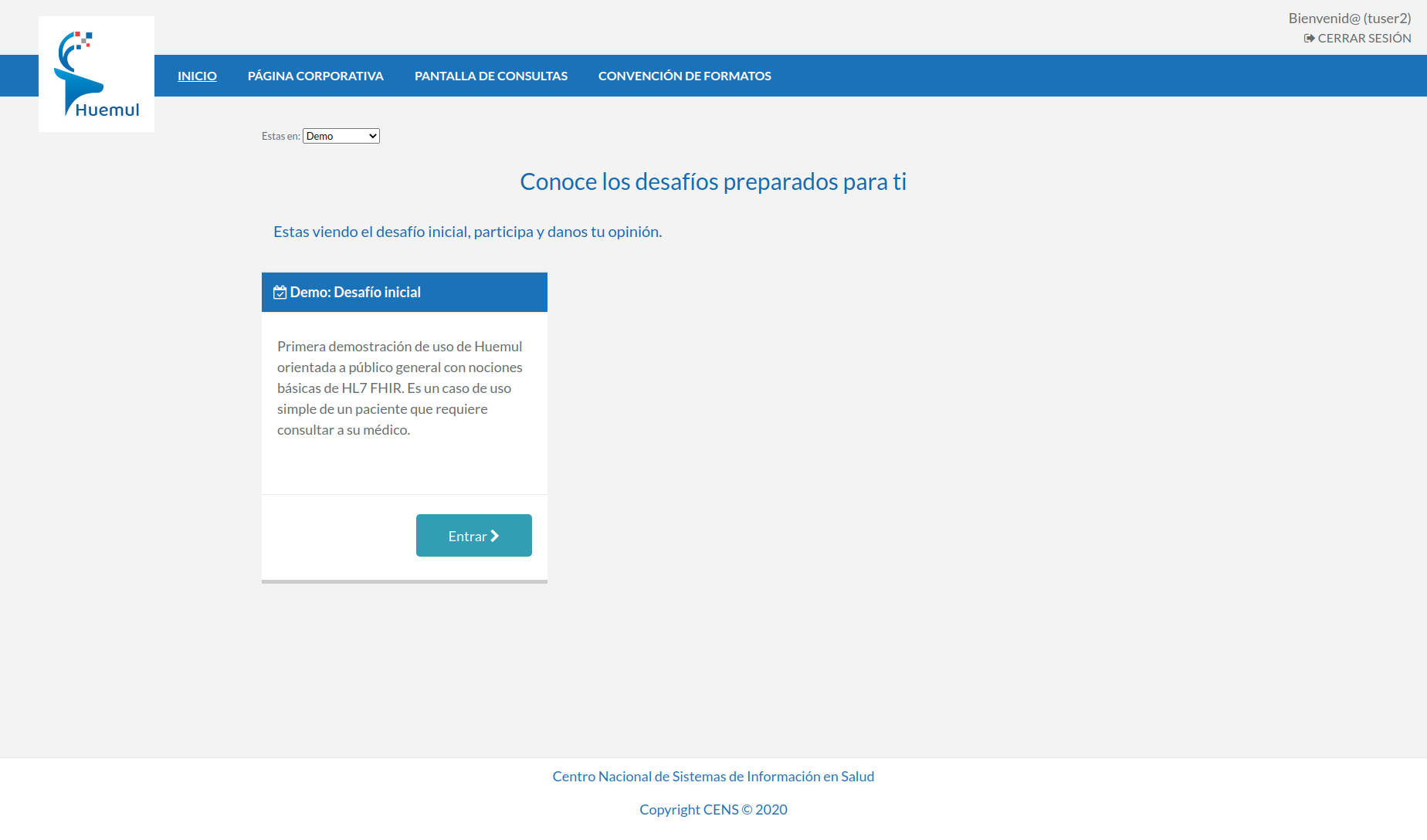

- You can display all tests or filter them by instance on this screen.
- The available tests will have a coloured button, while those not enabled will have a grey button.


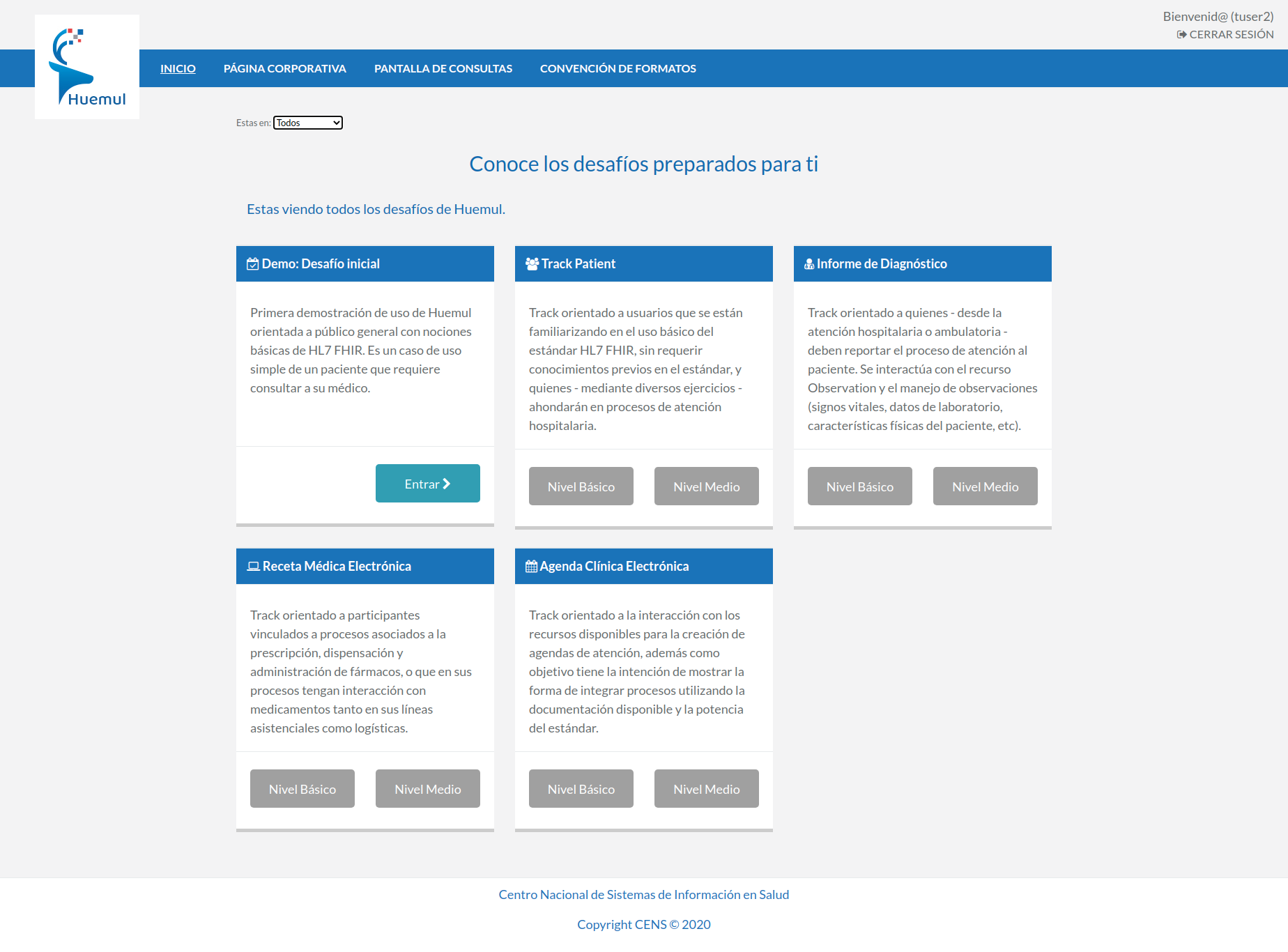


#

# Detail of the challenge or test

- You will find the scenario description and information relevant to the test.


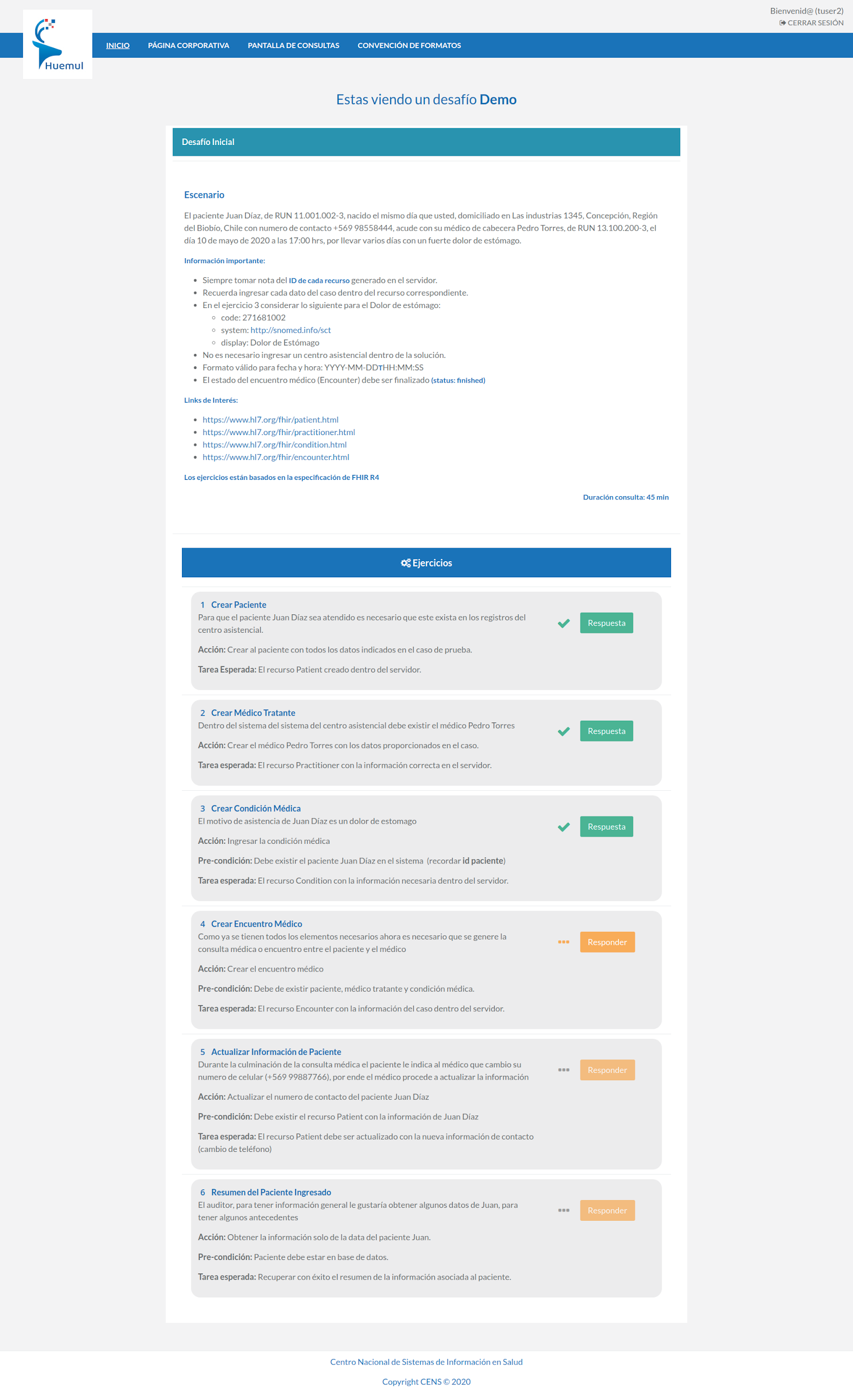


- Below is a list of the exercises to be answered.


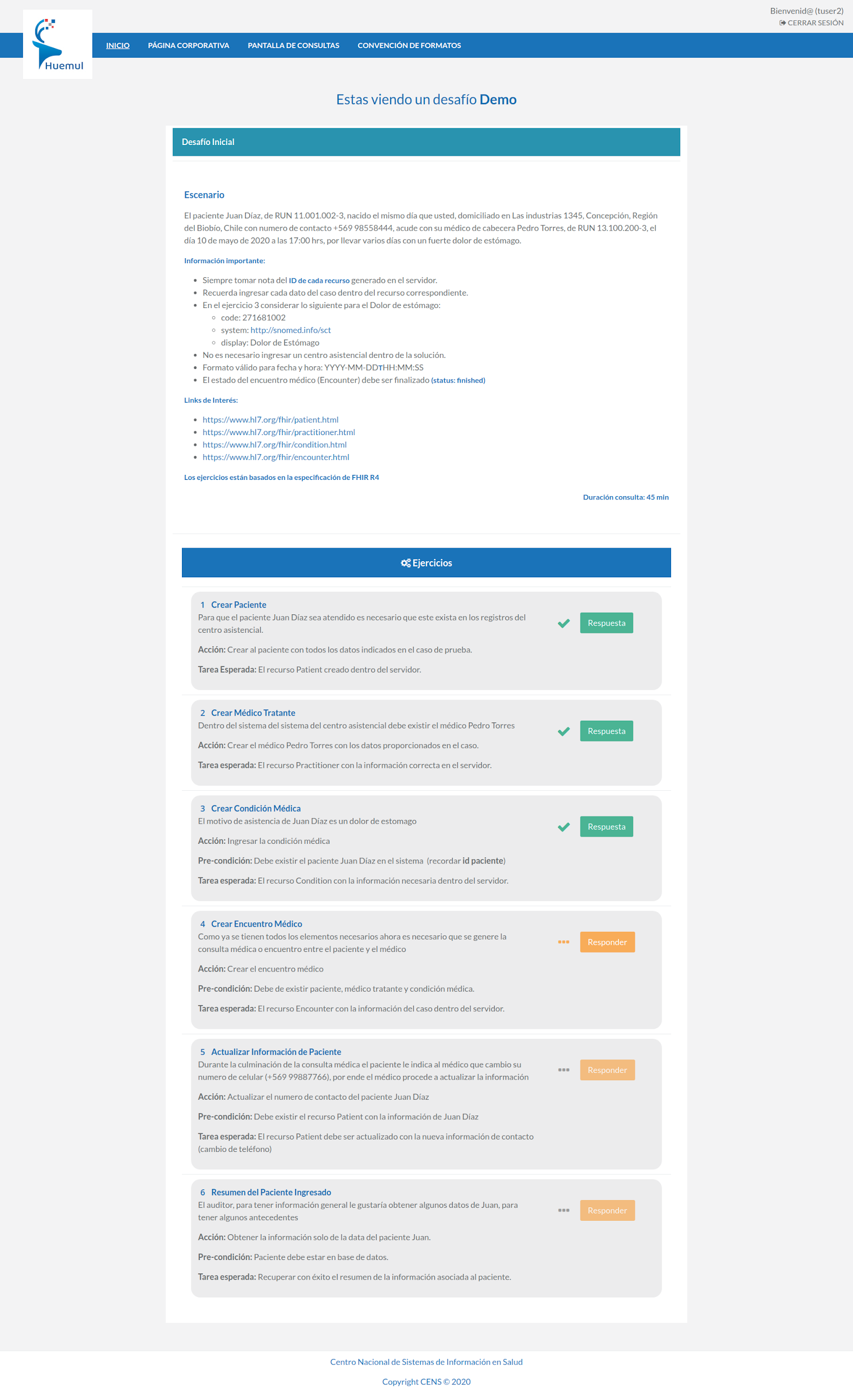


**> Orange Button:** Unanswered exercise

**> Green Button:**  Exercise with the correct answer

**> Red Button:** Exercise with the wrong answer

**Unanswered exercise**
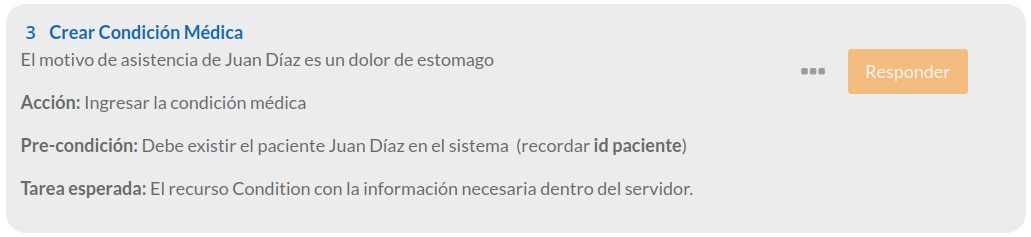


Al pinchar en el botón naranjo, se abrirá la página para enviar la respuesta.

## Ejercicio con respuesta correcta


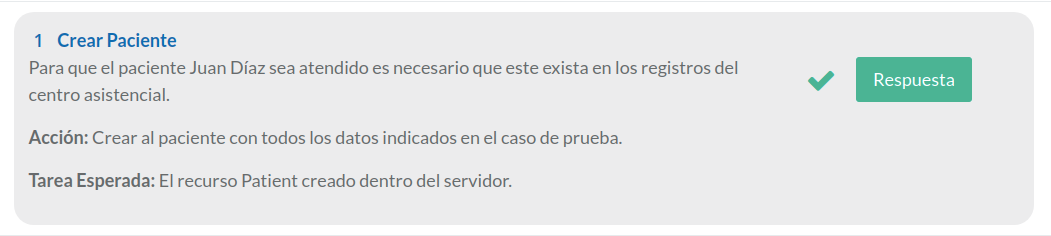


Clicking on the orange button will open the page to submit your response.

## Exercise with the wrong answer


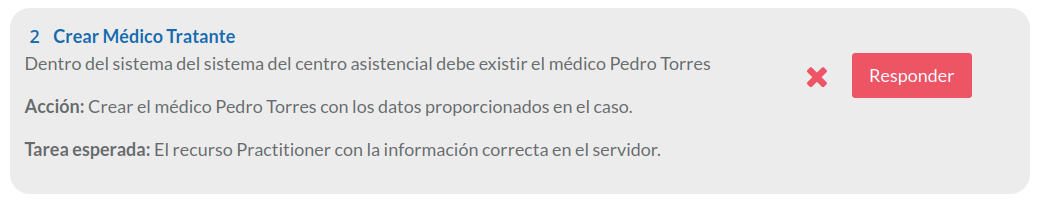


When you click on the red button, a modal will open with the details of the answer sent, and if it is incorrect, it allows you to answer again, so a button "Answer again" will open the page to send another answer.

#
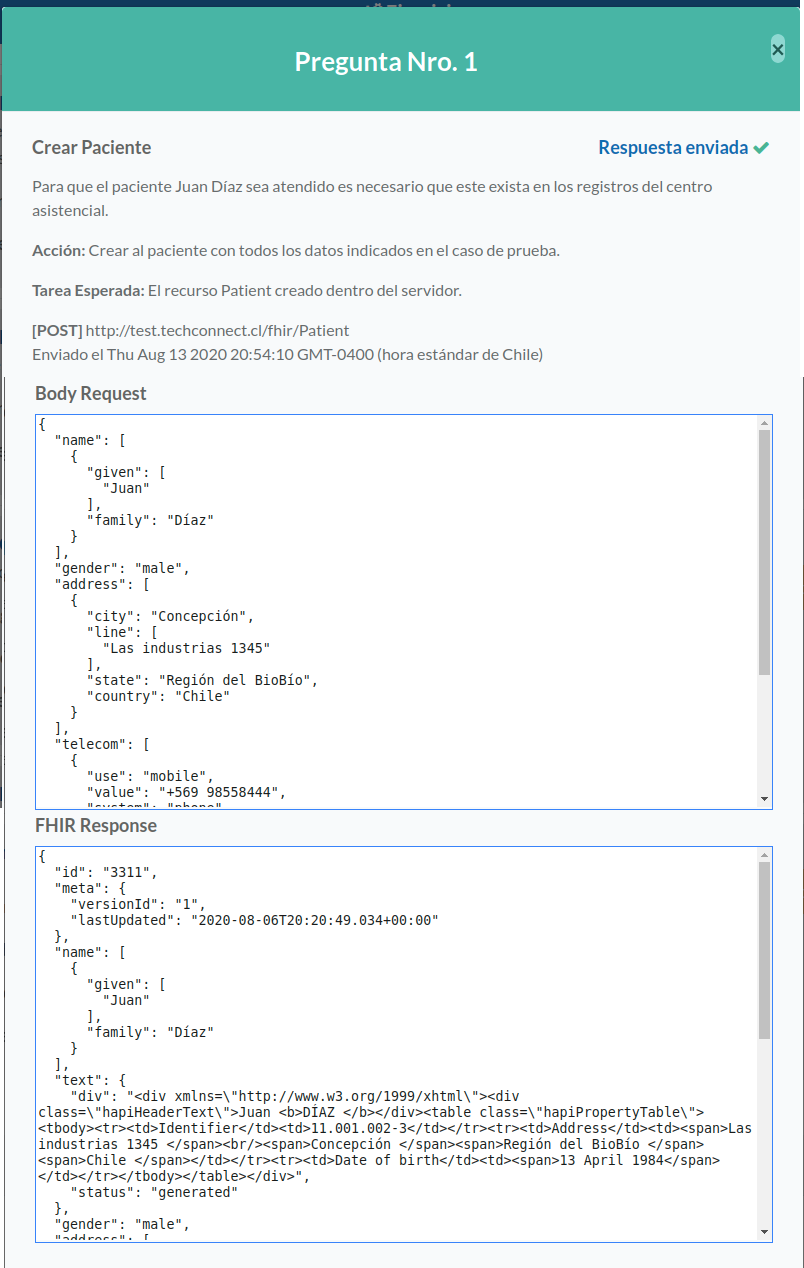
Correct answer modal

# Wrong answer modal

#
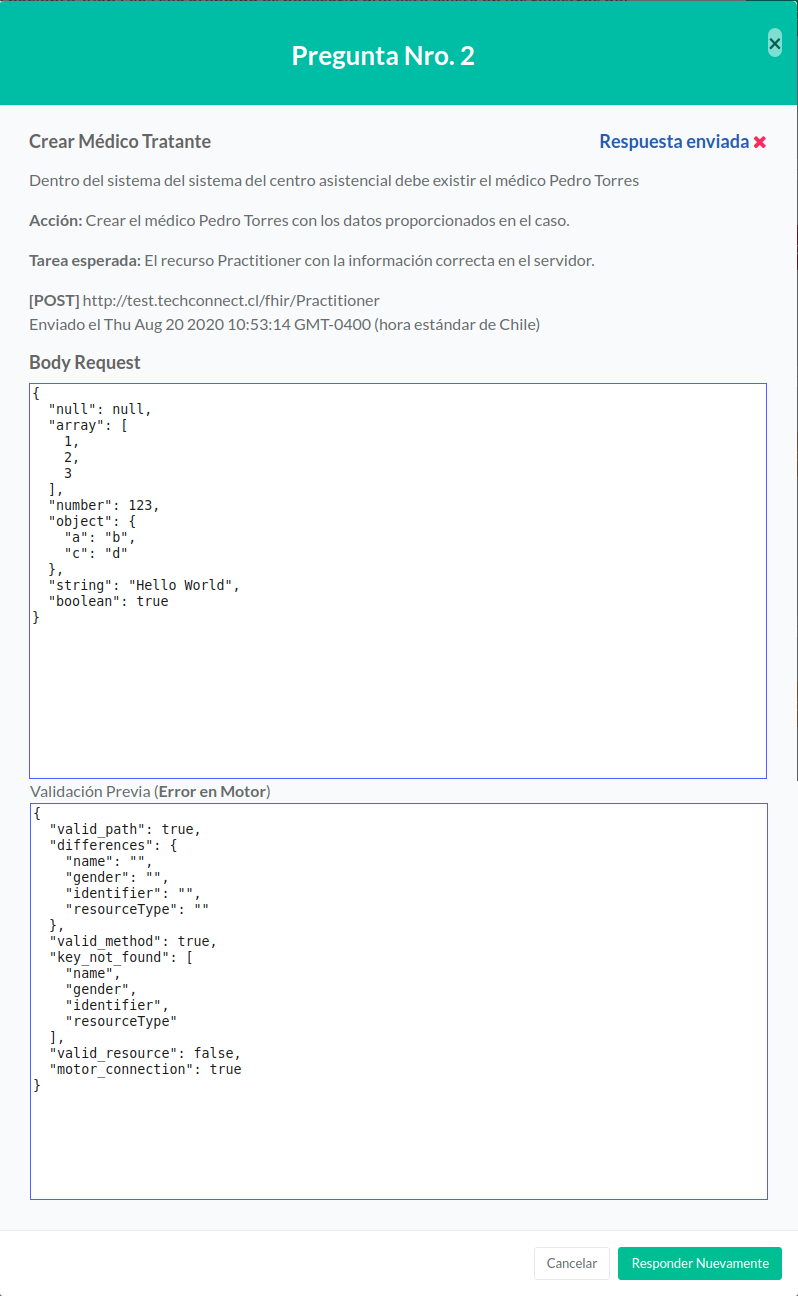


# Send Response

On the screen to send the answer, the question statement and essential information to answer (action, precondition, expected task, etc.) is displayed; there is also a button to visualise the description of the scenario, and below in notifications, feedback is given to the user to improve and correct the answers.


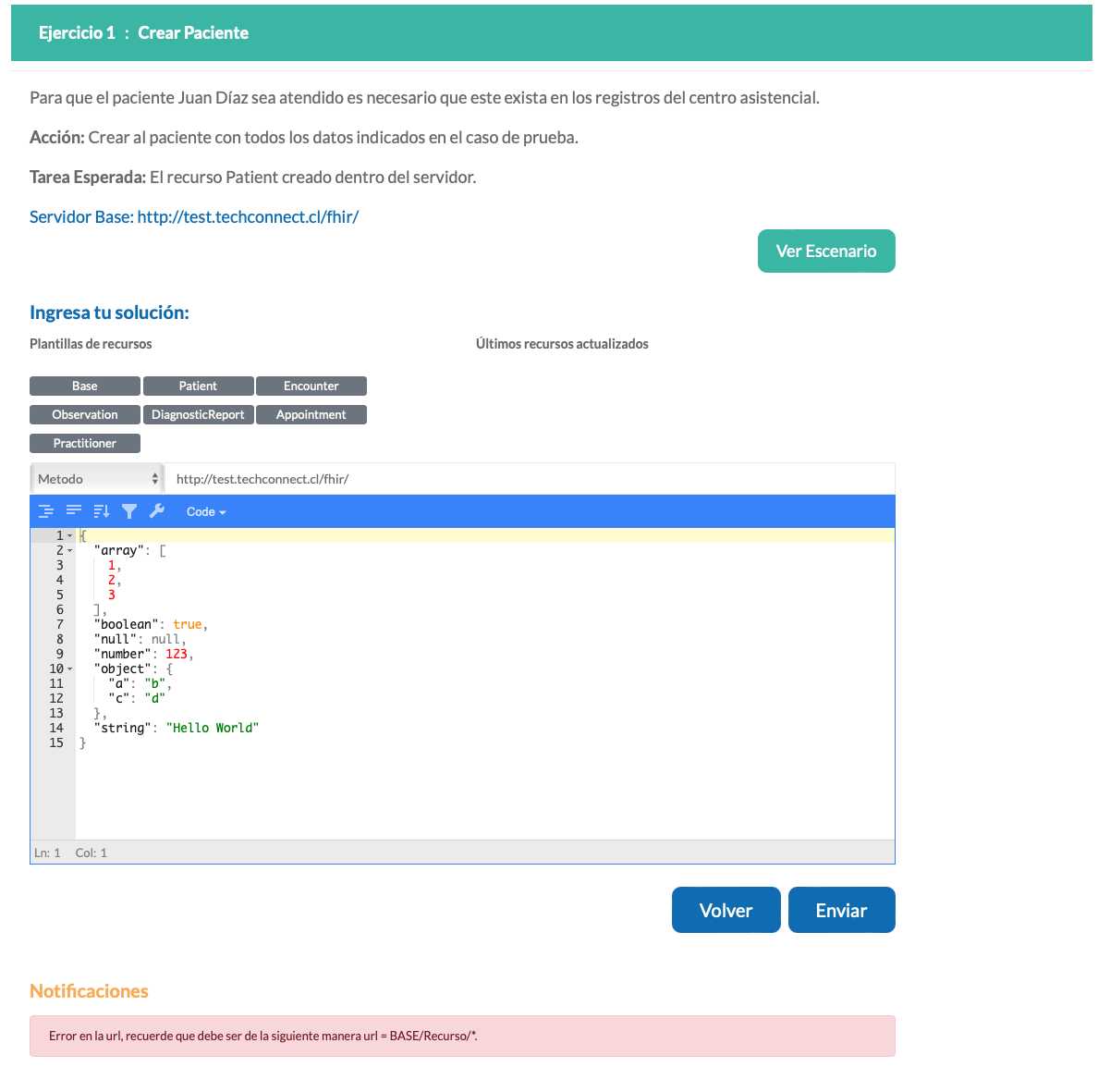


# Queries Screen

This screen is for searching the data of a given resource and server.


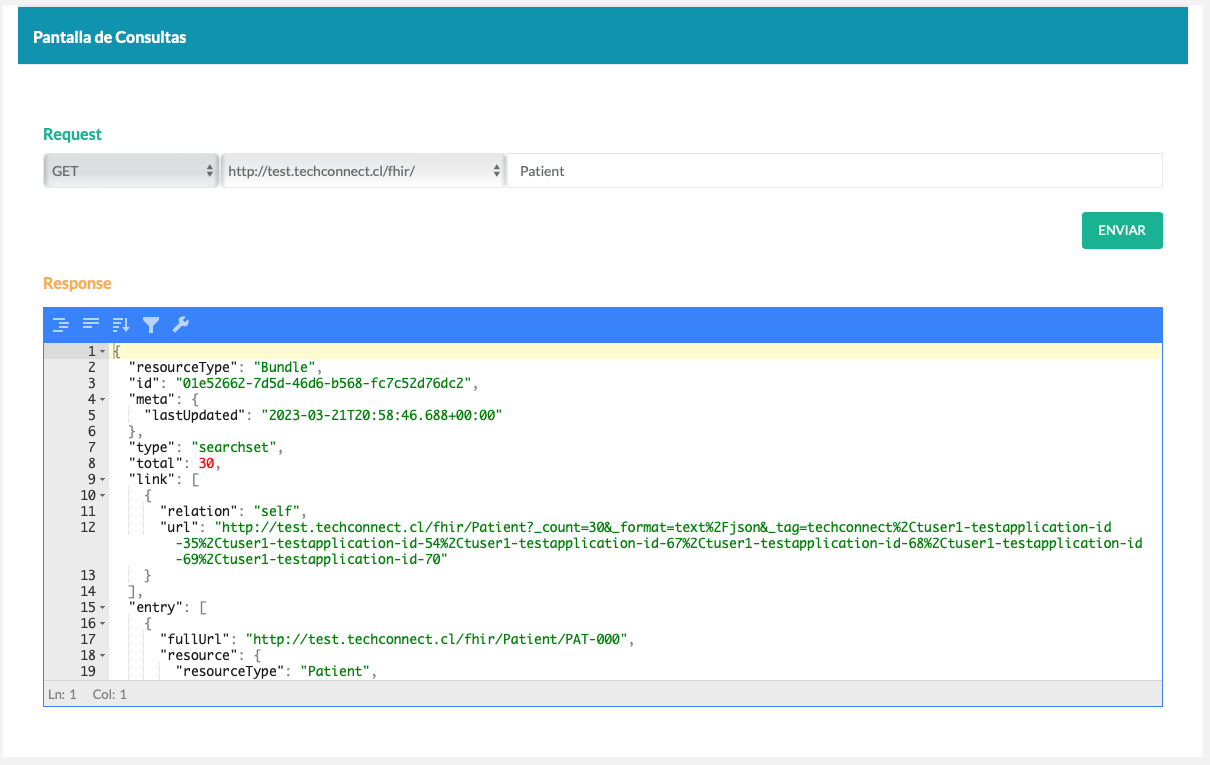

Supplement: Multimedia Appendix 2 [file mededu_v10i1e45413_app2.docx]
